# Genetic variation at the IL-18–137C>G is associated with poor sepsis prognosis and enhanced inflammatory responses: a multicenter hospital-based study

**DOI:** 10.3389/fgene.2026.1820930

**Published:** 2026-06-03

**Authors:** Zhuoji Li, Wanjie Gu, Lizhen Liu, Jiekai Li, Manting Zhang, Wanchun Yang, Meiting Yang, Jingqi Zhang, Haotian Zhong, Yuchun Liu, Junbing He, Haiyan Yin, Yiming Shao

**Affiliations:** 1 Jinan University, Guangzhou, Guangdong, China; 2 The Intensive Care Unit, The Second Affiliated Hospital, Guangdong Medical University, Zhanjiang, Guangdong, China; 3 The Intensive Care Unit, The First Dongguan Affiliated Hospital, Guangdong Medical University, Dongguan, Guangdong, China; 4 The Intensive Care Unit, Affiliated Hospital of Guangdong Medical University, Zhanjiang, Guangdong, China; 5 The Intensive Care Unit, Jieyang People’s Hospital, Jieyang, Guangdong, China

**Keywords:** IL-18, inflammation, polymorphism, prognosis, sepsis

## Abstract

**Background:**

Sepsis is a life-threatening condition caused by dysregulated immune responses, leading to inflammation, tissue damage, and organ failure. This study investigates the role of the IL-18 rs187238 (−137C>G) polymorphism in sepsis susceptibility, progression, and patient prognosis.

**Methods:**

A multicenter case-control study was conducted with 784 sepsis patients and 776 healthy controls. The IL-18 rs187238 polymorphism was genotyped using imLDR™ multiplex SNP genotyping method. ELISA and qRT-PCR were used to detect related inflammatory cytokine expression, while functional analysis was performed using dual-luciferase assays to evaluate the impact of the rs187238 variant on IL-18 promoter activity.

**Results:**

We observed a significant association between the rs187238 polymorphism and 28-day ICU mortality in sepsis patients. The CG/GG genotypes (OR = 1.470, 95% CI = 1.029–2.129, *P* = 0.037) were more frequently observed in non-survivors compared to survivors, with a notable difference in the frequency of the G allele (OR = 1.534, 95% CI = 1.111–2.133, *P* = 0.010). Kaplan-Meier survival analysis confirmed that patients with CG/GG genotypes had significantly lower 28-day survival rates compared to those with the CC genotype (*P* = 0.028). However, no significant differences in genotype and allele frequencies were observed between cases and healthy controls, nor between sepsis and septic shock patients. Sepsis patients with CG/GG genotypes had significantly higher IL-18 levels than those with the CC genotype. Dual-luciferase assays confirmed that the G allele increased IL-18 promoter activity, supporting its genetic influence on IL-18 expression. Additionally, sepsis patients with CG/GG genotypes expressed significantly increased levels of IL-1β, IL-6, and ICAM-1 than those with CC genotype. IL-18 treatment enhanced the expression of IL-1β, IL-6, IL-27, TNF-α, and MCP-1 in THP-1 macrophages upon LPS stimulation. In HUVECs, IL-18 treatment further enhanced LPS-induced IL-6, IL-27, TNF-α, and ICAM-1 expression, while promoting apoptosis and reducing VE-cadherin levels, emphasizing its role in inflammation and endothelial dysfunction in sepsis.

**Conclusion:**

The IL-18 rs187238 C>G polymorphism is linked to higher IL-18 expression and intensified inflammatory responses, which are associated with poor sepsis prognosis. The sepsis-associated risk rs187238-G allele serves as a potential prognostic biomarker for sepsis-related mortality. Targeting IL-18 or its genetic variations might offer new avenues for sepsis therapy.

## Introduction

Sepsis remains a leading cause of morbidity and mortality worldwide, particularly in critically ill patients. The condition is characterized by a dysregulated host immune response to infection, which triggers systemic inflammation, tissue damage, and organ failure. Despite significant advances in our understanding, sepsis continues to present a major challenge in intensive care units, with outcomes heavily influenced by early diagnosis, timely treatment, and patient-specific factors (Tirupakuzhi Vijayaraghavan and Adhikari, 2022; Martin-Loeches et al., 2024). In regions like Asia, healthcare disparities and delays in intervention contribute to higher mortality rates, further underscoring the need for better diagnostic and therapeutic strategies (Desposito and Bascara, 2024). The pathophysiology of sepsis involves intricate interactions between pathogens and the immune system, driving both acute and chronic immune dysfunction (Wu et al., 2024). Recent studies have highlighted the critical role of genetic factors in shaping the immune response, influencing the disease course, and determining patient prognosis (Darnifayanti et al., 2023; Hernandez-Beeftink et al., 2022). Understanding these genetic underpinnings holds promise for improving sepsis management, offering potential biomarkers for both early detection and tailored therapeutic interventions.

Interleukin-18 (IL-18) is a pro-inflammatory cytokine that plays a crucial role in immune regulation and the development of inflammatory diseases, including sepsis. It activates innate immune cells such as macrophages, dendritic cells, and natural killer (NK) cells, driving the production of inflammatory cytokines like IL-1β and TNF-α, which are key in sepsis-related inflammation (Ihim et al., 2022; Landy et al., 2024). Dysregulated IL-18 production contributes to tissue damage and organ dysfunction in sepsis (Herminghaus et al., 2025). Elevated IL-18 levels correlate with the severity of sepsis, making it a potential diagnostic and prognostic biomarker (Zheng, 2025; Zhou et al., 2024). Additionally, IL-18 interacts with other cytokines such as IL-1β and IL-17A, amplifying inflammation in sepsis (Cross, 2016). Beyond inflammation, IL-18 also regulates apoptosis, which worsens organ dysfunction and patient outcomes (Mierzchala-Pasierb et al., 2019). Recent studies highlight the role of the NLRP3-IL-1β/IL-18 signaling axis in sepsis progression, particularly in aging phenotypes (Xu et al., 2025). Moreover, targeting both IL-18 and IL-1 could offer therapeutic benefits in managing septic shock (Vanden et al., 2014a). IL-18’s involvement in sepsis pathophysiology makes it an attractive therapeutic target for reducing inflammation and improving outcomes.

Variants in the IL-18 promoter region influence cytokine levels and disease outcomes. The IL-18 rs187238 polymorphism plays a significant role in disease susceptibility, particularly in inflammatory conditions. For example, the rs187238 polymorphism is linked to changes in high-density lipoprotein (HDL) levels in COVID-19 outpatients, suggesting its potential as a marker for immune response modulation during viral infections (Viana et al., 2023). Additionally, a functional IL-18 polymorphism has been associated with the severity of bronchial asthma, highlighting its impact on immune-mediated disorders (Harada et al., 2009). IL-18 polymorphisms, including rs187238, also serve as genetic biomarkers in autoimmune diseases like systemic lupus erythematosus (Rezaieyazdi et al., 2025). In cancer research, the IL-18 rs187238 variant is associated with lung cancer susceptibility, further illustrating its role in immune response regulation (Chen et al., 2024). Moreover, studies have found links between IL-18 polymorphisms (rs187238 and -607C>A) and serum IL-18 levels in type 2 diabetes patients, suggesting its involvement in metabolic disorders (Razooqi et al., 2024). These findings underscore the critical role of IL-18 genetic variations in modulating immune responses across diverse inflammatory diseases.

This study, conducted across multiple hospitals, included 784 sepsis patients and 776 healthy controls to explore the clinical relevance of the IL-18 genetic variant, rs187238 C>G (−137C>G), in relation to sepsis susceptibility and prognosis for the first time. Through a series of follow-up experiments, we assessed the impact of the rs187238 polymorphism on IL-18 gene expression and the subsequent inflammatory responses triggered during sepsis. By examining these functional polymorphisms, we aim to identify potential genetic markers that could inform novel therapeutic approaches for sepsis, potentially leading to improved strategies for diagnosis, prognosis, and treatment of this life-threatening condition.

## Methods

### Study population

This study included patients who were admitted to the Intensive Care Units (ICU) of four major hospitals: Affiliated Hospital of Guangdong Medical University (Zhanjiang), Harbin Medical University Affiliated Fourth Hospital (Harbin), Jieyang People’s Hospital (Jieyang), and Wuhan Central Hospital (Wuhan), between May 2017 and November 2023. The diagnosis of sepsis was made by experienced clinicians in accordance with the Sepsis-3.0 diagnostic criteria (Singer et al., 2016). Sepsis patients who met these criteria were recruited for the study, and peripheral blood samples were collected within 12 h of sepsis diagnosis. Data collected from these patients included their age, gender, infection source, microbiological culture results, Sequential Organ Failure Assessment (SOFA) score, and Acute Physiology and Chronic Health Evaluation (APACHE) II score. Patients were excluded based on the following criteria according to our previous studies (He et al., 2025; He et al., 2018): 1) individuals under the age of 18; 2) pregnant or breastfeeding women; 3) patients diagnosed with conditions other than sepsis; 4) patients with hematological diseases, malignancies, COVID-19, or chronic illnesses; 5) individuals with long-term use of immunosuppressants or corticosteroids; 6) patients who had undergone organ transplantation. Healthy control subjects were selected from individuals undergoing routine health check-ups at the medical center. Volunteers were screened using questionnaires, physical examinations, and medical record analysis. Exclusion criteria for controls included: 1) any autoimmune diseases, infectious diseases, cancer, or hematological disorders; 2) individuals with organ dysfunction, coronary artery disease, atherosclerosis, hyperlipidemia, or metabolic disorders. The sepsis patients were classified into two subgroups according to the Sepsis-3.0 diagnostic criteria: 1) Sepsis group and 2) Septic Shock group. In addition, patients were further divided into two groups based on 28-day survival: 1) Survivor group, which includes patients who survived 28 days post-diagnosis of sepsis; and 2) Mortality group, which includes patients who died within 28 days of diagnosis. All study participants, both in the case and control groups, provided written informed consent, and the study was approved by the hospital ethics committees.

### Genotyping of IL-18 rs187238 polymorphism

Genotyping of IL-18 rs187238 Polymorphism was performed by using imLDR™ Multiplex SNP Genotyping Kit. DNA was extracted from peripheral blood samples using TIANamp DNA extraction kit (Tiangen Biotechnology, China). 1 μL of the DNA sample was loaded onto a 1% agarose gel for electrophoresis to check for quality and concentration. Based on the estimated concentration, the DNA samples were diluted to a working concentration of 5–10 ng/μL. A multiplex PCR reaction was used to detect the IL-18 rs187238 polymorphism. The PCR primers used were: rs187238F, TCA​GCA​AGC​TGG​GGA​GAG​AAT​G; rs187238R, GAA​GGC​ACA​GAG​CCC​CAA​CTT​T. The ligation reaction for the PCR products used specific primers as follows: rs187238RC: TTC​CGC​GTT​CGG​ACT​GAT​ATG​AGC​CCC​AAC​TTT​TAC​GGA​AGA -ACAG; rs187238RG: TAC​GGT​TAT​TCG​GGC​TCC​TGT​GAG​CCC​CAA​CTT​TTA​CGG​AAG​AAC​AC; rs187238RP: ATT​TCA​TGA​AAA​TAG​TGA​TAT​TAC​ATT​AAA​AGA​AGT​ACT​CTT​TTT​TTT​TTT​TTT​TTT. The ligation product (0.5 μL) was mixed with 0.5 μL Liz500 size standard and 9 μL Hi-Di formamide. The mixture was denatured at 95 °C for 5 min and then loaded onto the ABI3730XL sequencer for data collection. The sequencing data collected from the ABI3730XL sequencer were analyzed using the GeneMapper 4.1 software (AppliedBiosystems, USA) to determine the genotype of each sample. In addition, 10% of the samples were randomly selected for repeat genotyping as an independent validation set to assess genotyping quality.

### Isolation of peripheral blood mononuclear cells (PBMCs)

A total of 159 subjects (86 sepsis patients and 73 healthy controls) were randomly selected for isolation of PBMCs from peripheral blood samples. PBMCs were isolated using Lymphoprep™ (Axis-Shield PoCAS, Oslo, Norway) by density gradient centrifugation. The procedure was as follows: 1) Equal volumes of physiological saline and blood were mixed in a centrifuge tube and well-mixed; 2) Lymphoprep™ was added to a new centrifuge tube, and the mixture was gently layered along the tube wall; 3) The mixture was centrifuged at 800 rpm for 40 min at room temperature. PBMCs were located in the middle layer, which was carefully aspirated into a new tube. The PBMCs were diluted with an equal volume of saline, and centrifuged at 300 rpm for 15 min; 4) The supernatant was discarded, and 1 mL of RNA key™ reagent was added to the cell pellet for RNA extraction.

### RNA extraction and quantitative Real-Time PCR (qRT-PCR)

Total RNA was extracted from PBMCs using RNAkey™ reagent (Sevenbio, Beijing, China). The RNA concentration and purity were assessed using a Nanodrop spectrophotometer. Reverse transcription was performed using the SevenFast® Two Step RT&qPCR Kit* (Sevenbio, Beijing, China), and cDNA was synthesized according to the manufacturer’s instructions. The qRT-PCR was conducted using a 7,500 Real-Time PCR system (Applied Biosystems, Thermo Fisher Scientific, USA). PCR conditions were as follows: 1) Initial denaturation at 95 °C for 5 s, followed by annealing at 58 °C for 20 s, and extension at 72 °C for 33 s for a total of 40 cycles; 2) Relative gene expression was calculated using the 2^−ΔΔCT^ method, normalizing to β-actin expression. The primers used for target genes such as IL-1β, IL-6, TNF-α, IL-18 and IL-27, were listed in Table 1.

**TABLE 1:** Primer sequences used in quantitative real-time PCR.

Primers of gene		Human (5’-…-3′)
β-actin	Forward	TCC​CTG​GAG​AAG​AGC​TAC​GA
	Reverse	AGC​ACT​GTG​TTG​GCG​TAC​AG
IL-27	Forward	CGG​AGG​GAG​TTC​ACA​GTC​AG
	Reverse	CAG​GTG​AGA​TTC​CGC​AAA​GC
IL-1β	Forward	GAA​GCT​GAT​GGC​CCT​AAA​CA
	Reverse	GCA​TCT​TCC​TCA​GCT​TGT​CC
IL-6	Forward	GAA​AGC​AGC​AAA​GAG​GCA​CT
	Reverse	TTT​CAC​CAG​GCA​AGT​CTC​CT
IL-18	Forward	TCT​TCA​TTG​ACC​AAG​GAA​ATC​GG
	Reverse	TCC​GGG​GTG​CAT​TAT​CTC​TAC
TNF-α	Forward	CCT​CTC​TCT​AAT​CAG​CCC​TCT​G
	Reverse	GAG​GAC​CTG​GGA​GTA​GAT​GAG

### Dual-luciferase reporter assay for IL-18 promoter activity

Based on our initial findings, a 1500-bp promoter sequence of the IL-18 gene (from −1311 to +189 bp) containing the rs187238 C>G polymorphism (−137C>G) was cloned into the pGL3 luciferase reporter vectors (Promega, Madison, WI, USA). The IL-18 promoter gene fragment was synthesized using PCR amplification with the following primer sequences: IL18-MluI-F (forward): 5′-ATTACGC GTT​CTA​CAG​TTG​GAA​GGT​GAA​AAC-3′; IL18-XhoI-R (reverse): 5′-TTA​CTC​GAG​CTG​CGA​CAA​ATA​GTT​TGT​TGC​G-3′. The resulting constructs were transfected into 293T and THP-1 cells (Shanghai Institute of Cell Biology, Shanghai, China) using Lipofectamine 2000 (Invitrogen, USA) or GP-transfect-Mate reagent (GenePharma, China). After 48 h of transfection, the promoter activities were measured using the dual-luciferase reporter assay system (Promega, USA). The relative luciferase activities were quantified in a Synergy H1 full-featured enzyme marker (BioTek, USA).

### Enzyme-linked immunosorbent assay (ELISA)

ELISA was performed according to the manufacturer’s instructions for each specific ELISA kit. Plasma samples from both septic patients and healthy controls were analyzed for pro-inflammatory cytokines (e.g., IL-1β, IL-6, IL-18, TNF-α) and endothelial injury-related markers (e.g., ICAM-1, VCAM-1). The absorbance was measured at 450 nm using a Synergy H1 full-featured enzyme marker (BioTek, USA). Standard curves were generated, and cytokine concentrations were determined using the regression model.

### IL-18 and LPS treatment experiments

THP-1 cells were cultured in RPMI 1640 medium supplemented with 10% FBS. Recombinant human IL-18 was purchased from Proteintech (Cat No. HZ-1340). The cells were treated with either medium alone, IL-18 (50 ng/mL), LPS (500 ng/mL), or a combination of LPS and IL-18 (10, 50, or 200 ng/mL) for 12 h. At each time point, THP-1 cells were collected for qRT-PCR assays of cytokine expression. Human umbilical vein endothelial cells (HUVECs) were sourced from the Cell Resource Center at Peking Union Medical College, China. These cells were cultured in an endothelial cell medium. The cells were incubated with either medium alone, IL-18 (50 ng/mL), LPS (200 ng/mL), or a combination of LPS and IL-18 (10, 50, or 200 ng/mL) for 12 h. At each time point, HUVECs were collected for annexin V apoptosis analysis and cytokine measurements.

### Flow cytometry analysis of apoptosis in HUVECs

HUVEC apoptosis was assessed using the Annexin V-FITC/PI apoptosis detection kit and flow cytometry on a CytoFlex S Flow Cytometer (Beckman Coulter). Briefly, cells were harvested using trypsin, followed by centrifugation at 900 rpm for 3 min. The cell pellet was resuspended in PBS, then stained with 2.5 µL of Annexin V-FITC for 20 min in the dark. PI (2.5 µL) was added 5 min before analysis, and the cells were incubated for another 20 min. After adding 200 µL of 1× Binding Buffer, the samples were analyzed for apoptosis.

### Statistical analyses

Statistical analyses were conducted using GraphPad Prism 8.0 (GraphPad Software Inc., San Diego, CA, USA) and SPSS Statistics 26 (IBM, NY, USA). To compare genotype and allele frequencies between groups, the Chi-square test or Fisher’s exact test was used. Kaplan–Meier survival curves were plotted to analyze the impact of IL-18 polymorphisms on sepsis mortality. A multivariable logistic regression analysis was conducted to assess the association between IL-18 polymorphisms and sepsis, adjusting for age, gender, illness severity (as indicated by APACHE II and SOFA scores), and other potential confounding variables. The Benjamini–Hochberg method was applied for genetic association testing of multiple comparisons to adjust for false discovery rate (FDR). This approach adjusts p-values to control FDR, helping to identify significant associations while decreasing the risk of type I errors. Power analysis using QUANTO 1.2 (QUANTO 1.2; University of Southern California, LA, USA) indicated that, with a significance level of 0.05 and an assumed odds ratio of 1.5, the study had 82.3% power to detect genotype-related differences for rs187238 based on the sample size. For two separate samples, either Student’s t-test or Mann-Whitney U test was used. All data are presented as mean ± standard error of the mean (SEM). P < 0.05 was considered statistically significant.

## Results

### Clinical characteristics of the study population

A total of 784 patients with sepsis and 776 healthy controls were included in this analysis. The characteristics of the participants are summarized in Table 2. Among the sepsis cohort, 53.6% had sepsis, and 46.4% had septic shock. The control group had a mean age of 51.1 ± 11.9 years, with 62.3% male participants. The sepsis patients had an average age of 61.0 ± 16.9 years, with 66.7% male.

**TABLE 2:** Clinical characteristics of sepsis patients and healthy controls.

Variable	Sepsis (n = 784)	Control (n = 776)	P value
Demographics			
Age, years, mean ± SD	61.0 ± 16.9	51.1 ± 11.9	<0.001
Gender, male/female, n	523/261	484/292	0.073
Sepsis status, n (%)			
Sepsis subtype	420 (53.6)	N.A	​
Septic shock	364 (46.4)	N.A	​
Source of infection, n (%)			
Respiratory tract infection	493 (62.9)	N.A	​
Abdominal infection	163 (20.8)	N.A	​
Primary bloodstream infection	119 (15.2)	N.A	​
Urinary tract infection	75 (9.6)	N.A	​
Catheter-associated infection	50 (6.4)	N.A	​
Trauma	49 (6.3)	N.A	​
Central nervous system	48 (6.1)	N.A	​
Others	42 (5.4)	N.A	​
Pathogenic bacteria, n (%)			
*Acinetobacter* baumannii	197 (25.1)	N.A	​
*Pseudomonas aeruginosa*	89 (11.4)	N.A	​
*Escherichia coli*	87 (11.1)	N.A	​
*Candida* albicans	79 (10.1)	N.A	​
*Klebsiella pneumoniae*	70 (8.9)	N.A	​
*Staphylococcus aureus*	64 (8.2)	N.A	​
Yeast	44 (5.6)	N.A	​
Stenotrophomonas maltophilia	45 (5.7)	N.A	​
Aspergillus	36 (4.6)	N.A	​
*Staphylococcus* epidermidis	12 (1.5)	N.A	​
Others	110 (14.0)	N.A	​
APACHE II score, mean ± SD	25.67 ± 5.80	N.A	​
SOFA score, mean ± SD	8.43 ± 4.34	N.A	​
28-day mortality, n (%)	229 (29.2)	N.A	​

N.A: not applicable; APACHE II: Acute Physiology and Chronic Health Evaluation II; sepsis-related organ dysfunction assessment (SOFA); Continuous data are expressed as the mean ± SD.

The most frequent infection source in sepsis patients was the respiratory system (493 cases, 62.9%), followed by abdominal infections (163 cases, 20.8%), bloodstream infections (119 cases, 15.2%), and urinary tract infections (75 cases, 9.6%). A variety of other infection sources, including catheter-associated infections (50 cases, 6.4%), trauma (49 cases, 6.3%), and intracranial infections (48 cases, 6.1%), were observed as well. Regarding pathogens, *Acinetobacter* baumannii was the predominant causative agent (197 cases, 25.1%), followed by *Pseudomonas aeruginosa* (89 cases, 11.4%), *Escherichia coli* (87 cases, 11.1%), and *Candida* albicans (79 cases, 10.1%). *Klebsiella pneumoniae*, *Staphylococcus aureus*, and several opportunistic pathogens also contributed significantly to the infection burden, with 110 cases (14.0%) attributed to other microorganisms. Some patients had multiple infection sites or were infected with multiple pathogens. The 28-day mortality rate among sepsis patients was 29.2%, with the mean APACHE II score being 25.67 ± 5.80 and the mean SOFA score being 8.43 ± 4.34.

### Genetic impact of IL-18 rs187238 polymorphism on sepsis onset and progression

The distribution of genotypes for IL-18 rs187238 in sepsis patients and healthy controls was consistent with Hardy-Weinberg equilibrium (P > 0.05). When comparing the genotype and allele frequencies of the IL-18 rs187238 C>G polymorphism between sepsis patients and controls, no significant differences were observed (All P > 0.05; Table 3), suggesting that this polymorphism is not associated with sepsis susceptibility. Further subgroup analysis revealed no notable differences in genotype or allele frequencies between the sepsis and septic shock groups (P = 0.161 for genotype, P = 0.118 for allele; Table 4). After adjusting for age, sex, SOFA, and APACHE II score by using a multivariable logistic regression analysis, the differences remained statistically non-significant. These findings imply that the IL-18 rs187238 polymorphism is unlikely to influence the development of septic shock.

**TABLE 3:** Genotype and allele frequencies distribution of IL-18 rs187238 polymorphism in the sepsis patients and healthy controls.

IL-18 rs187238	Control n (%)	Sepsis n (%)	*P*	Adjusted *P* ^a^	Odds ratio (95% CI)	Adjusted *P* ^b^	Adjusted odds ratio (95% CI)^b^
CC	586 (75.5)	619 (79.0)	0.226	0.301	-	-	-
CG	178 (22.9)	157 (20.0)	-	-	-	-	-
GG	12 (1.6)	8 (1.0)	-	-	-	-	-
CC + CG vs. GG	764 (98.5)	776 (99.0)	0.378	0.378	1.524 (0.641, 3.576)	0.300	1.660 (0.637, 4.325)
CG + GG vs. CC	190 (24.5)	165 (21.0)	0.105	0.210	0.822 (0.649, 1.039)	0.064	0.788 (0.613, 1.014)
C	1350 (87.0)	1395 (89.0)	-	-	1.000 (References)	-	-
G	202 (13.0)	173 (11.0)	0.089	0.210	0.829 (0.668, 1.031)	-	-

OR: odds ratio; 95% CI: 95% confidence interval.

^a^ False discovery rate-adjusted P-value for multiple hypotheses testing using the Benjamini–Hochberg method.

^b^ Adjusted for age, and gender by using multivariable logistic regression analysis.

**TABLE 4:** Genotype and allele frequencies distribution of IL-18 rs187238 polymorphism between sepsis subtype and septic shock.

IL-18 rs187238	Sepsis subtype n (%)	Septic shock n (%)	*P*	Adjusted *P* ^a^	Odds ratio (95% CI)	Adjusted *P* ^b^	Adjusted odds ratio (95% CI)^b^
CC	339 (80.7)	280 (76.9)	0.161	0.194	-	-	-
CG	79 (18.8)	78 (21.4)	-	-	-	-	-
GG	2 (0.5)	6 (1.7)	-	-	-	-	-
CC + CG vs. GG	419 (99.5)	357 (98.4)	0.154	0.194	0.286 (0.058, 1.188)	0.821	0.843 (0.191, 3.726)
CG + GG vs. CC	81 (19.3)	84 (23.1)	0.194	0.194	1.256 (0.894, 1.765)	0.690	1.079 (0.742, 1.571)
C	758 (90.1)	637 (87.6)	-	-	1.000 (References)	-	-
G	82 (9.9)	91 (12.4)	0.118	0.194	1.287 (0.941, 1.765)	-	-

OR: odds ratio; 95% CI: 95% confidence interval.

^a^ False discovery rate-adjusted P-value for multiple hypotheses testing using the Benjamini–Hochberg method.

^b^ Adjusted for age, gender, APACHE II, and SOFA, scores by using multivariable logistic regression analysis.

### Genetic effects of IL-18 rs187238 polymorphism on sepsis mortality

**FIGURE 1:**
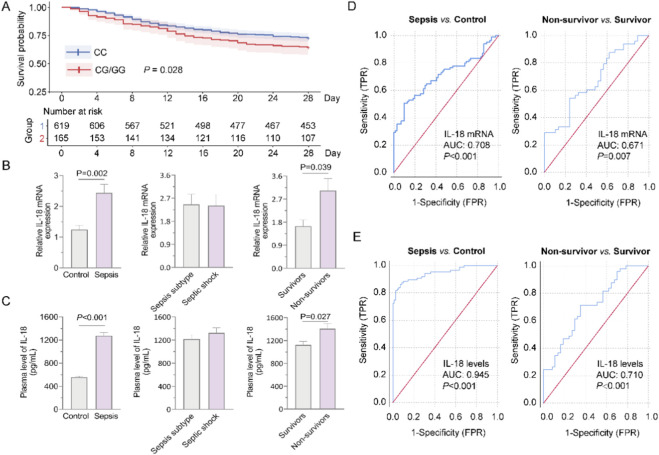
IL-18 gene expression levels in healthy controls and sepsis patients. **(A)** Kaplan-Meier survival analysis of IL-18 rs187238 and its impact on 28-day survival in sepsis patients; **(B)** qRT-PCR analysis comparing IL-18 gene expression between healthy controls (n = 73) and sepsis patients (n = 86), as well as between sepsis subtype (n = 37) and septic shock (n = 49) group, and between survivors (n = 38) and non-survivors (n = 48); **(C)** ELISA analysis of IL-18 plasma levels between healthy controls and sepsis patients, as well as between sepsis subtypes and the septic shock group, and between survivors and non-survivors; **(D)** ROC analysis of IL-18 gene expression using these sepsis and healthy samples to assess diagnostic performance **(E)** ROC analysis of IL-18 plasma levels using these sepsis and healthy samples to assess diagnostic performance. Error bars represent the standard error of the mean (SEM).

We explored the potential association between the IL-18 rs187238 polymorphism and 28-day ICU mortality in sepsis patients by analyzing genotype and allele frequencies in various patient groups. As presented in Table 5, our findings showed that the frequency of the CG/GG genotypes and the G allele was significantly higher in non-survivors compared to survivors (CC vs. CG + GG: P = 0.037, OR = 1.470, 95% CI 1.029–2.129; C allele vs. G allele: P = 0.010, OR = 1.534, 95% CI 1.111–2.133). Similar results were also observed in a multivariable logistic regression analysis, adjusted for age, sex, SOFA, and APACHE II score (CC vs. CG + GG: P = 0.042, OR = 1.479, 95% CI 1.015–2.154). These results suggested that IL-18 rs187238 C>G polymorphism may be linked to an increased risk of mortality in sepsis patients. Additionally, Kaplan-Meier survival analysis (Figure 1A) indicated that patients with the rs187238 CG/GG genotypes had a significantly lower 28-day survival rate compared to those with the CC genotype (P = 0.028). This suggests that the IL-18 rs187238 C>G polymorphism may serve as a potential prognostic factor in sepsis, with CG/GG genotypes potentially increasing mortality risk. The G allele is associated with more severe outcomes in sepsis, highlighting its clinical relevance.

**TABLE 5:** Genotype and allele frequencies distribution of IL-18 rs187238 polymorphism between surviving and non-surviving septic patients.

IL-18 rs187238	Survivors n (%)	Non-survivors n (%)	*P*	Adjusted *P* ^a^	Or (95% CI)	Adjusted *P* ^b^	Adjusted odds ratio (95% CI)^b^
CC	449 (80.9)	170 (74.2)	0.005	0.013	-	-	-
CG	104 (18.7)	53 (23.2)	-	-	-	-	-
GG	2 (0.4)	6 (2.6)	-	-	-	-	-
CC + CG vs. GG	553 (99.6)	223 (97.4)	0.010	0.013	0.134 (0.028, 0.560)	0.016	0.134 (0.026, 0.685)
CG + GG vs. CC	106 (19.1)	59 (25.8)	0.037	0.037	1.470 (1.029, 2.129)	0.042	1.479 (1.015, 2.154)
C	1002 (90.3)	393 (85.8)	-	-	1.000 (References)	-	-
G	108 (9.7)	65 (14.2)	0.010	0.013	1.534 (1.111, 2.133)	-	-

OR: odds ratio; 95% CI: 95% confidence interval.

^a^ False discovery rate-adjusted P-value for multiple hypotheses testing using the Benjamini–Hochberg method.

^b^ Adjusted for age, gender, APACHE II, and SOFA, scores by using multivariable logistic regression analysis.

### IL-18 expression in sepsis patients and its clinical diagnostic value

To investigate IL-18 expression, we analyzed peripheral blood samples from the randomly selected 86 sepsis patients (CC = 68; CG + GG = 18) and 73 healthy controls (CC = 55; CG + GG = 18) using qRT-PCR. No difference in genotype distribution compared to the overall cohort. As shown in Figure 1B, IL-18 mRNA levels were significantly higher in sepsis patients compared to controls. There was no significant difference in IL-18 mRNA between sepsis and septic shock subgroups (P > 0.05). However, IL-18 mRNA levels were notably higher in the 28-day ICU non-survivor group than the survivor group (P = 0.039). Figure 1C demonstrates significantly higher serum IL-18 levels in sepsis patients (P < 0.001) compared to controls. No difference was found between sepsis and septic shock subgroups (P > 0.05), but serum IL-18 levels were significantly higher in the non-survivor group (P = 0.027). The IL-18 gene expression model had an AUC of 0.708 for distinguishing sepsis patients from controls (Figure 1D). In contrast, the serum IL-18 protein model performed better, with an AUC of 0.945 for identifying sepsis. The serum-based diagnostic model for predicting 28-day ICU mortality showed an AUC of 0.710 (Figure 1E), highlighting its superior diagnostic value over gene expression.

### IL-18 rs187238 C>G polymorphism and its association with IL-18 expression in sepsis patients and healthy controls

**FIGURE 2:**
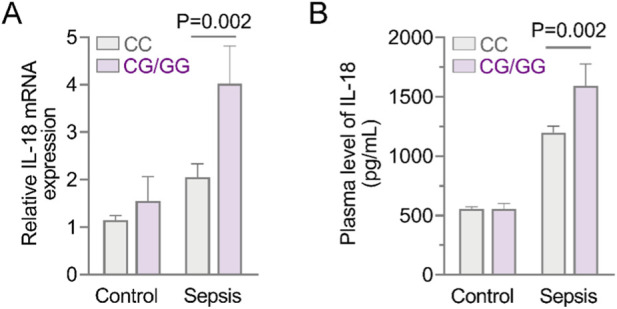
IL-18 expression in sepsis patients and healthy controls with different IL-18 rs187238 polymorphisms. **(A)** Comparison of IL-18 gene expression between different genotypes of IL-18 rs187238 polymorphism in both sepsis patients and healthy controls; **(B)** Comparison of IL-18 plasma levels between different genotypes of IL-18 rs187238 polymorphism in both sepsis patients and healthy controls. CC (n = 55) and CG/GG (n = 18) in control; CC (n = 68) and CG/GG (n = 18) in sepsis; Error bars represent standard error of the mean (SEM).

In the SNP subgroup analysis, we found that sepsis patients with the CG/GG genotypes had significantly higher levels of IL-18 mRNA compared to those with the CC genotype (P = 0.022, Figure 2A). In contrast, no significant differences in IL-18 mRNA expression were observed between the different genotypes in the healthy control group. Similarly, when assessing IL-18 serum protein levels through ELISA, we observed that the CG/GG genotypes in sepsis patients were associated with significantly higher serum IL-18 levels compared to the CC genotype (P = 0.022, Figure 2B). However, no such differences were found in the healthy controls between the different rs187238 genotypes.

### Influence of IL-18 rs187238 polymorphism on pro-inflammatory cytokine expression

**FIGURE 3:**
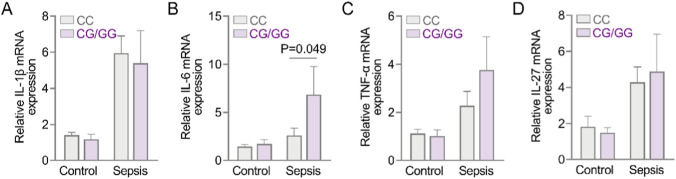
Pro-inflammatory cytokine mRNA expression levels in sepsis patients and healthy controls with different IL-18 rs187238 polymorphisms. Randomly selected peripheral blood mononuclear cells (PBMCs) from 86 sepsis patients and 73 healthy controls were analyzed by qRT-PCR. **(A-D)** Distribution of IL-1β, IL-6, TNF-α, and IL-27 expression between different rs187238 genotypes in healthy controls and sepsis patients. CC (n = 55) and CG/GG (n = 18) in control; CC (n = 68) and CG/GG (n = 18) in sepsis; Error bars represent standard error of the mean (SEM).

To assess how the IL-18 rs187238 polymorphism affects pro-inflammatory cytokine expression, we analyzed the gene expression levels of several cytokines in PBMCs from 86 sepsis patients and 73 healthy controls. As shown in Figure 3, qRT-PCR analysis revealed significantly elevated IL-16 expression in sepsis patients with the rs187238 CG/GG genotypes compared to those with the CC genotype (P = 0.049). Although TNF-α levels were also higher in patients with the CG/GG genotypes, this difference was not statistically significant. No substantial differences in IL-1β and IL-27 expression were detected between the genotypes.

**FIGURE 4:**
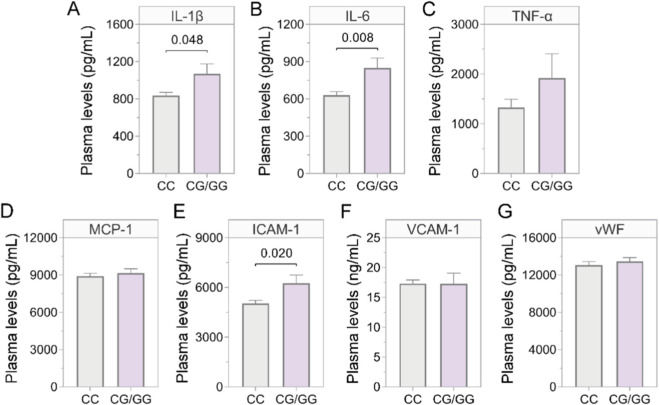
Plasma concentrations of pro-inflammatory cytokines and endothelial injury-related factors in sepsis patients with different IL-18 rs187238 polymorphisms. Plasma samples from 86 sepsis patients (n = 68 for CC, n = 18 for CG/GG) were analyzed by ELISA for the concentrations of cytokines **(A-G)** IL-1β, IL-6, TNF-α, MCP-1, ICAM-1, VCAM-1, and vWF based on different rs187238 genotypes. Error bars represent standard error of the mean (SEM).

Subsequently, we measured the concentrations of key pro-inflammatory cytokines (IL-1β, IL-6, TNF-α, MCP-1) and endothelial damage markers (ICAM-1, VCAM-1, vWF) in the plasma of sepsis patients. Figure 4 illustrates that plasma levels of IL-1β (P = 0.048), IL-6 (P = 0.008), and ICAM-1 (P = 0.020) were significantly higher in patients with the rs187238 CG/GG genotypes compared to those with the CC genotype. Although plasma TNF-α was elevated in patients with CG/GG genotypes, no significant difference was found. Additionally, the concentrations of MCP-1, VCAM-1, and vWF in plasma did not significantly vary between the different genotypes.

### Functional consequences of the IL-18 rs187238C>G polymorphism on IL-18 expression

**FIGURE 5:**
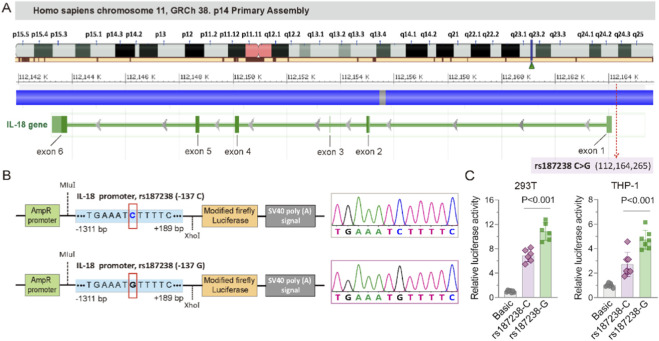
Location of the IL-18 rs187238C>G polymorphism in IL-18 gene and its effect on the transcriptional regulation of IL-18 expression. **(A)** The rs187238 C>G (−137C>G) polymorphism is located in the transcriptional promoter region of the IL-18 gene; **(B)** The IL-18 promoter region spanning from −1311bp to +189bp, with either the C or G allele at the rs187238 locus, was inserted into pGL3 luciferase reporter vectors for further investigation; **(C)** 293T and THP-1 cells were transfected with plasmids containing the C or G allele constructs, and after 48 h, promoter activity was assessed using a dual-luciferase reporter assay. Error bars represent the standard error of the mean (SEM).

The genetic analysis indicated that sepsis patients with the CG/GG genotypes exhibit higher IL-18 expression. As shown in Figure 5A, the rs187238 C>G variation is located within the −137 bp region of the IL-18 promoter, which likely impacts transcriptional regulation. To further explore the impact of this polymorphism, we created a dual-luciferase reporter plasmid with the rs187238 C-to-G variant and transfected it into 293T and THP-1 cells for functional assessment (Figure 5B). The results presented in Figure 5C show that cells with the −137G allele (rs187238-G) demonstrated significantly higher IL-18 promoter activity compared to those containing the −137C allele (rs187238-C) (P < 0.001). These results suggest that the rs187238 C>G polymorphism enhances IL-18 transcriptional activity, which may lead to increased IL-18 expression, contributing to the pathophysiology of sepsis.

### IL-18 enhances LPS-induced pro-inflammatory cytokine expression and cell apoptosis

**FIGURE 6:**
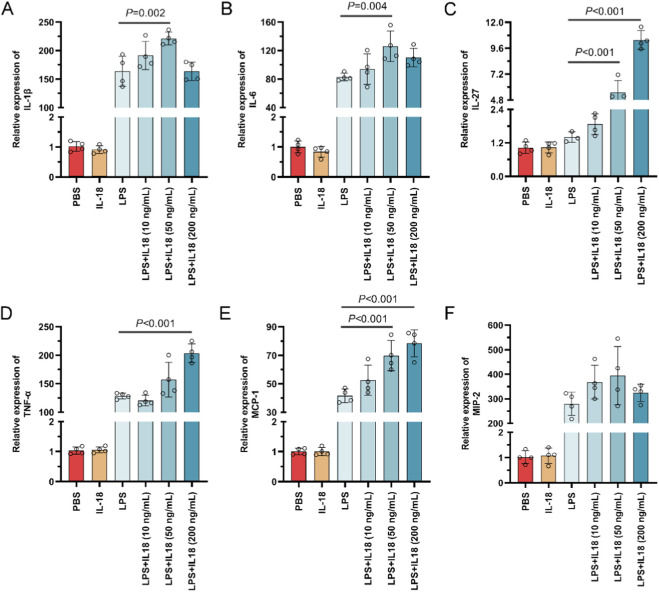
IL-18 potentiates LPS-induced expression of pro-inflammatory cytokines in THP-1 macrophages. The expressions of IL-1β **(A)**, IL-6 **(B)**, IL-27 **(C)**, TNF-α **(D)**, MCP-1 **(E)**, and MIP-2 **(F)** by qRT-PCR in THP-1 cells incubated with either medium alone, IL-18 (50 ng/mL), LPS (500 ng/mL), or a combination of LPS and IL-18 (10, 50, or 200 ng/mL) for 12 h. The data are presented as bar graphs representing the mean ± SD, based on at least three independent experiments, each performed in triplicate.

We assessed the impact of IL-18 on pro-inflammatory cytokine expression in THP-1 macrophages upon LPS stimulation, as presented in Figure 6. Treatment with 50 ng/mL IL-18, followed by LPS (500 ng/mL), significantly increased IL-1β, IL-6, IL-27, and MCP-1 expression compared to LPS alone. Increasing the IL-18 dose to 200 ng/mL further enhanced the LPS-induced expression of IL-27, TNF-α, and MCP-1, though IL-18 had no significant effect on MIP-2 expression.

**FIGURE 7:**
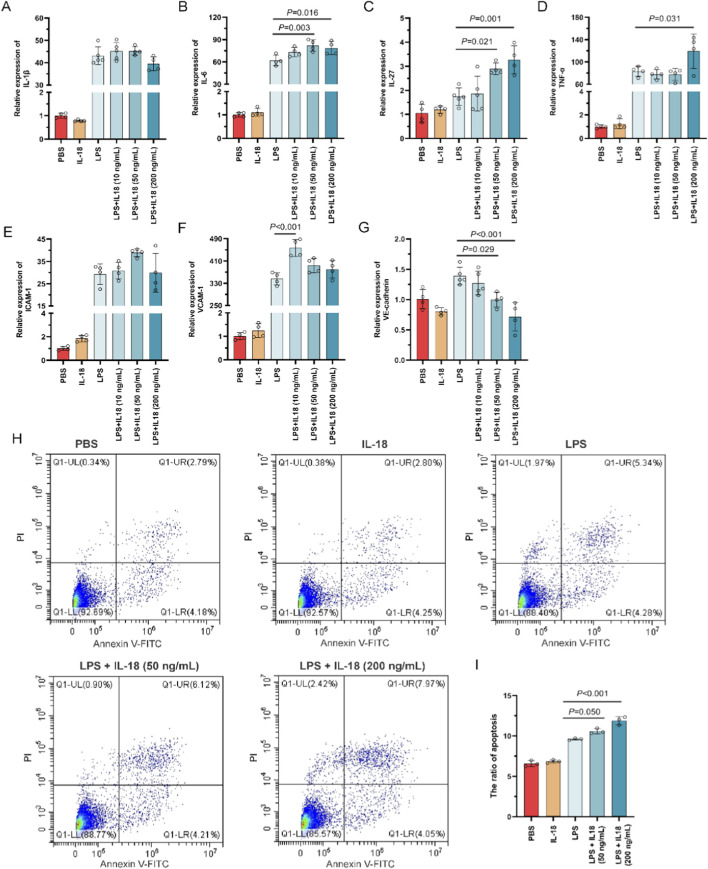
IL-18 enhances lipopolysaccharide (LPS)-induced pro-inflammatory cytokine expression and apoptosis in HUVECs. The expression of IL-1β **(A)**, IL-6 **(B)**, IL-27 **(C)**, TNF-α **(D)**, ICAM-1 **(E)**, VCAM-1 **(F)**, and VE-cadherin **(G)** by qRT-PCR from HUVECs incubated with either medium alone, IL-18 (50 ng/mL), LPS (200 ng/mL), or a combination of LPS and IL-18 (10, 50, or 200 ng/mL) for 12 h **(H,I)** Apoptosis analysis of HUVECs treated with medium, IL-18 (50 ng/mL), LPS (200 ng/mL), or a combination of LPS and IL-18 (50, or 200 ng/mL) for 12 h using annexin-V-FITC/propidium iodide (PI) staining and flow cytometry. Bar graphs show the mean ± SD for at least three independent experiments, each performed in triplicate.

We also evaluated the effects of IL-18 on cytokine expression and cell apoptosis in HUVECs upon LPS stimulation. 50 ng/mL IL-18 enhanced LPS-induced IL-6, IL-27, and ICAM-1 expression, while reducing VE-cadherin levels (Figures 7A–G). At 200 ng/mL, IL-18 further amplified IL-6, IL-27 and TNF-α expression but continued to suppress VE-cadherin. Notably, IL-18 did not significantly affect LPS-induced IL-1β levels. In terms of apoptosis, LPS treatment alone significantly increased apoptosis (Figures 7H,I), while co-treatment with LPS and 200 ng/mL IL-18 resulted in a substantial increase in apoptosis, indicating that IL-18 potentiates LPS-induced endothelial cell apoptosis.

## Discussion

Recent research has consistently shown the dysregulation of immune-inflammatory responses plays a pivotal role in the onset and progression of adverse outcomes in sepsis patients (Lin et al., 2024; Zhang et al., 2025; Wang et al., 2025). Findings from our previous studies, as well as those of other research groups, have emphasized the significance of single nucleotide polymorphisms (SNPs) in pro-inflammatory genes, which influence both the immune response and an individual’s susceptibility to sepsis and its progression (He et al., 2025; He et al., 2018; Xu et al., 2023; He et al., 2022; Chen et al., 2021). In this study, we explored the association between sepsis and the functional promoter polymorphism rs187238 C>G in the IL-18 gene. Our results indicate a notable correlation between the rs187238 risk-G allele and an upregulation of IL-18 expression, which in turn enhances inflammatory responses. This genetic variation is associated with worse sepsis outcomes, including an increased risk of mortality, further supporting the role of IL-18 in sepsis pathophysiology.

IL-18, a key pro-inflammatory cytokine, plays a crucial role in regulating immune responses, particularly in inflammatory diseases such as sepsis. Our findings revealed significantly elevated IL-18 mRNA and serum levels in sepsis patients compared to healthy controls, consistent with prior studies underscoring IL-18’s involvement in sepsis pathogenesis (Herminghaus et al., 2025; Zheng, 2025). Notably, the non-survivor group (28-day ICU mortality) had significantly elevated IL-18 expression levels compared to survivors, further suggesting that IL-18 may serve as an important prognostic marker for sepsis severity (Wu et al., 2019). The diagnostic value of IL-18 was also examined, with our analysis revealing that the serum IL-18 protein model outperformed the gene expression model in distinguishing sepsis patients from healthy controls, with an AUC of 0.945 for serum IL-18 levels. This suggests that serum IL-18 may offer superior diagnostic value, particularly for early identification of sepsis (Papatheodorou et al., 2025; Wynn et al., 2016). In addition, the serum IL-18 model showed an AUC of 0.710 for predicting 28-day ICU mortality. These findings support a role for IL-18 in sepsis diagnosis and prognosis, suggesting its potential as a biomarker for early detection and risk stratification. However, its clinical utility should be interpreted with caution, as the current results are based on a single cohort without external validation and lack comparisons with established biomarkers (e.g., procalcitonin and C-reactive protein); therefore, these findings should be considered preliminary and require validation in larger, independent cohorts.

The IL-18 rs187238 polymorphism has been implicated in various inflammatory conditions, with several studies suggesting its role in immune regulation and disease outcomes. For instance, Viana et al. reported that the rs187238 variant was linked to changes in high-density lipoprotein (HDL) levels in COVID-19 outpatients, underscoring its potential in modulating immune responses (Viana et al., 2023). Similarly, Mazurek-Mochol et al. observed alterations in IL-18 gene expression in gingival tissue of periodontitis patients, highlighting its involvement in inflammatory diseases (Mazurek-Mochol et al., 2022). Gupta et al. also identified an association between this polymorphism and susceptibility to tuberculosis in a North Indian cohort, demonstrating its broader relevance in infectious diseases (Gupta et al., 2025). Moreover, the work by Magri et al. pointed out the clinical implications of IL-18 polymorphisms in systemic lupus erythematosus, particularly the rs187238 (−137G>C) variant, which was linked to disease severity and susceptibility (Magri et al., 2025). In the present study, we focused on the genetic association between the rs187238 polymorphism and sepsis, specifically its impact on sepsis mortality. Our findings revealed that the CG/GG genotypes and the G allele were significantly more frequent in non-survivors compared to survivors. Kaplan-Meier survival analysis confirmed that patients carrying the CG/GG genotypes exhibited notably lower 28-day survival rates than those with the CC genotype. These results suggest that the rs187238 C>G polymorphism may serve as a potential prognostic marker for mortality risk in sepsis. However, no significant association was observed between the polymorphism and sepsis onset or progression, indicating its more prominent role in sepsis prognosis rather than in the initial development of the condition.

We further assessed the functional impact of the IL-18 rs187238 C>G polymorphism on IL-18 expression. Previous studies have shown that variations in the IL-18 gene, particularly the rs187238 polymorphism, can alter IL-18 gene expression, with some reports suggesting that carriers of the G allele exhibit elevated IL-18 levels. For instance, Hoseini et al. observed that individuals with the GG genotype exhibited significantly higher IL-18 expression compared to those with the GC/CC genotypes, which was also linked to coronary artery disease development (Hoseini et al., 2018). Similarly, research by Yue et al. found that the GG genotype was associated with increased IL-18 expression and higher susceptibility to idiopathic recurrent miscarriage (Yue et al., 2015). Our findings align with these observations, as sepsis patients carrying the G allele showed enhanced IL-18 expression levels. Additionally, Zhang et al. supported these results, demonstrating a positive correlation between the G allele of rs187238 and elevated IL-18 levels, with implications for ischemic stroke risk (Zhang et al., 2010). These findings are further corroborated by Anyona et al., who highlighted that IL-18 polymorphisms, including rs187238, can affect immune responses and disease susceptibility (Anyona et al., 2011). To explore the functional effects of the rs187238 polymorphism, we performed luciferase assays to investigate the impact of the rs187238 C>G variant on IL-18 gene promoter activity. The results confirmed that the sepsis-associated G allele significantly increased IL-18 promoter activity. Additionally, our study revealed that sepsis patients carrying the CG/GG genotypes expressed significantly higher levels of IL-18 compared to those with the CC genotype, underscoring the role of this polymorphism in modulating regulation of IL-18 expression during sepsis. In contrast, no such difference was observed between the CC and CG/GG genotypes in healthy individuals. These findings suggest that the genetic effect of the rs187238 C>G variation is more pronounced in the context of sepsis, pointing to the potential role of this SNP in influencing the prognosis of sepsis rather than its onset.

IL-18, a key pro-inflammatory cytokine, plays a pivotal role in driving the production of inflammatory mediators such as TNF-α, IL-1β, and IL-6, which are essential contributors to the systemic inflammation observed in sepsis. Studies have highlighted that the effects of IL-18 are primarily regulated through interactions with several key signaling pathways, especially NF-κB and MAPK (Hiraki et al., 2012). For example, Yoo et al. demonstrated that IL-18 induces MCP-1 production via the PI3K/Akt and MEK/ERK pathways in macrophages (Yoo et al., 2005), while Faggioni et al. showed that IL-18 binding protein can mitigate LPS-induced lethality, reinforcing IL-18’s central role in inflammatory responses during sepsis (Faggioni et al., 2001). In line with these findings, our study observed that IL-18 treatment significantly enhanced the expression of pro-inflammatory cytokines, including IL-1β, IL-6, IL-27, TNF-α, and MCP-1 in THP-1 macrophages following LPS stimulation. In HUVECs, IL-18 treatment further elevated IL-6, IL-27, TNF-α and ICAM-1 expression, while also promoting apoptosis and reducing VE-cadherin levels. These results underscore the critical role of IL-18 in the inflammatory processes of sepsis. We also assessed the effect of the rs187238 polymorphism on inflammatory responses by analyzing cytokine and endothelial marker levels in sepsis patients and healthy controls. Our results showed that sepsis patients carrying the G allele of rs187238 had significantly higher levels of IL-1β, IL-6, and ICAM-1, suggesting that the G allele amplifies the inflammatory response in sepsis. These findings support the hypothesis that the sepsis-associated G allele of rs187238 enhances IL-18 promoter activity, leading to increased IL-18 gene transcription and subsequently heightened inflammation, which may contribute to worse clinical outcomes in sepsis. These observations align with previous studies demonstrating the crucial role of IL-18 in sepsis-related inflammation. Vanden Berghe et al. highlighted the importance of IL-18 as a therapeutic target for protecting against septic shock (Vanden et al., 2014b), and Mi et al. further supported this by showing that IL-18 can drive inflammatory signaling (Mi et al., 2022). Additionally, Li et al. identified a novel anti-IL-1R7 antibody that reduces IL-18-mediated inflammatory signaling, providing a potential therapeutic strategy for mitigating sepsis-induced inflammation (Li et al., 2021). Together with our findings, these studies underscore the potential of targeting IL-18 or its genetic variants for improving sepsis management and patient prognosis. However, our study did not explore the detailed mechanistic pathways, such as transcription factor binding or downstream signaling pathways (e.g., NF-κB activation), which are important for fully understanding the role of the IL-18 rs187238 C>G polymorphism in sepsis-associated inflammation. Future studies are warranted to investigate these mechanisms and further elucidate the biological effects of IL-18 and its genetic variants in sepsis.

This study has several limitations that should be addressed. First, the broad inclusion criteria and the use of regional central referring hospitals for participant recruitment may have introduced selection bias. Second, the use of healthy controls instead of critically ill controls aimed to ensure a homogeneous control group and minimize confounding from other diseases, though it may limit the applicability of our findings to critically ill patients. Third, while the sample size is adequate for preliminary analysis, it remains relatively small and consists solely of Chinese Han individuals, which may limit the generalizability of the findings to other ethnic groups. Therefore, larger studies with more diverse populations are necessary to confirm and extend our findings. Fourth, although we sought to reduce heterogeneity by excluding patients with specific underlying diseases, the presence of other comorbidities in sepsis patients could still impact the genetic findings and contribute to variability in this case-control analysis. Fifth, while the focus was on the rs187238 polymorphism, other functional polymorphisms, either within IL-18 or related to inflammatory pathways, may also influence the expression and activity of IL-18. Subgroup analyses by infection source, pathogen type, or treatment were not performed, which may also influence IL-18 levels and clinical outcomes, representing a study limitation. Additionally, although the IL-18 rs187238 polymorphism is associated with sepsis prognosis, its modest effect size and borderline significance suggest that further research is needed to confirm its clinical relevance as a reliable prognostic marker. Investigating the effects of such polymorphisms in future studies could provide a more comprehensive understanding of the genetic basis of sepsis susceptibility and progression.

## Conclusion

In this study, we present evidence for the first time linking the IL-18 rs187238 C>G polymorphism with sepsis outcomes in a Chinese Han population. The sepsis-associated rs187238 G allele was found to significantly upregulate IL-18 expression, enhancing the production of pro-inflammatory cytokines and contributing to the exacerbation of the inflammatory response. This genetic variation was strongly associated with increased 28-day ICU mortality, indicating that the G allele serves as a potential prognostic marker for sepsis-related mortality. Our findings suggest that IL-18 plays a central role in the progression of sepsis and its clinical outcomes. These results may help explain the variability in sepsis prognosis and could inform future therapeutic strategies targeting IL-18 to improve patient outcomes.

## Data Availability

The original contributions presented in the study are included in the article/Supplementary Material, further inquiries can be directed to the corresponding authors.
