# Supplementary material for: Genetic variation at the IL-18–137C>G is associated with poor sepsis prognosis and enhanced inflammatory responses: a multicenter hospital-based study
Source: Front Genet. 2026 Jun 3;17:1820930. doi: 10.3389/fgene.2026.1820930 (PMC13271677; doi:10.3389/fgene.2026.1820930)
Supplement: Supplementary file 1 [file Table1.docx]

**Additional file 1:** Genotype distributions of Hardy-Weinberg equilibrium.

| **IL-18 polymorphism** | **Patients n (%)** | **Controls n (%)** | **Sepsis subtype n (%)** | **Septic shock n (%)** | **Survivors n (%)** | **Non-survivors n (%)** |
| --- | --- | --- | --- | --- | --- | --- |
| **rs187238** |  |  |  |  |  |  |
| CC | 433 (62.4) | 447 (64.1) | 339 (80.7) | 280 (76.9) | 449 (80.9) | 170 (74.2) |
| CG | 233 (33.6) | 225 (32.3) | 79 (18.8) | 78 (21.4) | 104 (18.7) | 53 (23.2) |
| CGG | 28 (4.0) | 25 (3.6) | 2 (0.5) | 6 (1.7) | 2 (0.4) | 6 (2.6) |
| Hardy-Weinberg equilibrium  test *P* value | 0.631 | 0.611 | 0.250 | 0.833 | 0.116 | 0.451 |
